# Possible link between familial susceptibility to cancer and the level of oxidative stress in thyroid cancer patients

**DOI:** 10.1186/s13053-024-00287-3

**Published:** 2024-08-23

**Authors:** Ivane Javakhishvili, Kote Mardaleishvili, Maka Buleishvili, Maia Mantskava, Irakli Chkhikvishvili, Sophio Kalmakhelidze, Nina Kipiani, Tamar Sanikidze

**Affiliations:** 1 Tbilisi State Medical University, Tbilisi, Georgia; 2 Department of Physics, Biophysics, Biomechanics and Informative Technologies of Tbilisi, State Medical University, Tbilisi, Georgia; 3 Caucasus International University, Tbilisi, Georgia; 4 Beritashvili Center of Experimental Biomedicine, Tbilisi, Georgia; 5 Beritashvili Center of Experimental Biomedicine, Tbilisi State Medical University, Tbilisi, Georgia

## Abstract

**Background:**

Hereditary cancer is estimated to account for up to 10% of the worldwide cancer burden; 5% of all thyroid cancers are thought to be genetic. Inheritance of a deleterious mutation in genes associated with a high lifetime risk of developing cancer. Cancer-predisposing genes can promote the initiation and progression of thyroid cancer by enhancing the activation of major signaling pathways through oxidative stress mechanisms.

**Aim:**

Identification of the possible link between familial susceptibility to cancer and the level of oxidative stress in thyroid cancer patients.

**Methods:**

Patients with thyroid cancer (with and without genetic predisposition) were investigated. Study participants were treated in Limited Liability Company (LLC) “Oncology Scientific Research Center” (Tbilisi, Georgia). The study group was collected between 2020 and 2021. In patients’ blood, the thyroid hormones content (free Triiodothyronine (fFT3), free Thyroxine (fFT4), bound Triiodothyronine (FT3), bound Thyroxine (FT4), Thyroid-stimulating hormone (TSH)), and oxidative stress intensity (total activity of non-enzymatic antioxidant system (TAA) and the lipid peroxidation product, malondialdehyde (MDA), content) were investigated.

**Results:**

The difference in free and bound forms of T3 and T4 levels in the blood serum between patients with thyroid cancer (Group 2 and Group 3) and the control group (Group 1) was not statistically significant (*F*_*1,2*_=0.5, *p*_*1,2*_=0.8, *F*_*1,3*_=2.31, *p*_*1,3*_=0.16). In patients with thyroid cancer the TSH level significantly increased compared to the control group (Group 1) (TSH (mean ± Std error): Group 1– 1.21 ± 0.12, Group 2–2.45 ± 0.11 (*F*_*1,2*_=107, *p*_*1,2*_<0.001), Group 3–2.47 ± 0.17 (*F*_*1,3*_=150, *p*_*1,3*_<0.001)) and the MDA levels increased by 4–5 fold. In patients with thyroid cancer from families with cancer aggregation(Group 2), the level of TAA statistically significantly decreased (*F*_*1 − 2*_*=*200; *p*_*1 − 2*_*<*0.001), in patients without genetic predisposition to cancer(Group 3), the level of TAA did not change compared to the control *(F*_*1 − 3*_*=* 2.13; *p*_*1 − 3*_*=*0.15),

**Conclusions:**

Oxidative stress plays a critical role in tumorigenesis, and antioxidant/oxidant imbalance may contribute to the malignant transformation of normal tissue. In patients with familial susceptibility to cancer mutations of several genes, which are involved in the regulation of oxidative metabolism, may contribute to the disruption of the redox balance, increase the level of oxidative stress, and contribute to the development of thyroid cancer.

## Background

The mortality rate caused by oncological diseases ranks second globally [1]. There were an estimated 18.1 million cancer cases around the world in 2020 (9.3 million cases were in men and 8.8 million in women), and thyroid cancer has occurred in 586,22 cases (3.2%). The thyroid tumor is the most common endocrine tumor [2] with an incidence of about 10.1 per 100,000 women and 3.1 per 100,000 men [3, 4]. In Georgia, according to the data of the National Center for Disease Control and Public Health of the Ministry of Labor, Health and Social Protection of Georgia, the incidence of malignant tumors is in second place and the number of new cases of diagnosed malignant tumors shows an increasing trend [5].

Thyroid cancer forms when the DNA in thyroid cells mutates and grows out of control, forming a tumor. The specific causes of thyroid cancer in an individual patient are not always clear. Malignant neoplasms arise due to genetic apparatus damage in germinal or somatic cells, making these cells sensitive to the effects of environmental carcinogenic factors that can activate the process of malignancy. These factors include hereditary conditions, gender, age, low iodine diet, exposure to radiation, including certain medical treatments, as well as fallout from nuclear weapons or power plant accidents, being overweight or obese, an enlarged thyroid, or a history of thyroiditis [6].

Hereditary cancer is estimated to account for up to 10% of the worldwide cancer burden; 5% of all thyroid cancers are thought to be genetic [7]. Many cancer-predisposing genes are involved in maintaining genome integrity and regulation balance between cell growth and cell death. There are about 500 known cancer-causing genes with reported mutations in somatic or germline DNA. From those genes, about 100 are mutated in the germline DNA of humans and could be inherited from parents to their children and predispose them to hereditary cancers. Inheritance of a deleterious mutation in genes confers a high lifetime risk of developing cancer [8]. In the case of prostate or breast cancer, the risk of the same location tumor diagnosed in the second generation is 30–40% [9]. Familial susceptibility to cancer is likely to be due to a combination of genetic and environmental influences [6].

According to Bano G, et al. (2021) [10], the genes, associated with thyroid cancer are protooncogenes *BRAF*, *RET*, *RAS*, *KRAS*, *NRAS*, sodium-iodide symporter SLC5A5 mediating active I-uptake in the thyroid, tumor suppressors *PTEN*, *PRKAR1A* [11], and *CHEK2* [12]. These gene mutations increase proliferation (*KRAS*, *NRAS*) [13], reduce apoptosis of tumor cells (RAS) [13], promote proto-oncogenes fusion (RET, PRKAR1A) that leads to uncontrolled cell growth and cancer [14], cause loss of ability to stop cell division and ensure cell restoration/destruction after damaged or strands break of the cell’s DNA (*CHEK2*) [12]. *BRAF* mutation could elicit strong inflammatory responses and is a significant source of ROS [14]. Oncogenic mutations in thyroid cancer could also increase the production of ROS through non-inflammatory mechanisms. Oncogene-induced production of ROS promotes the initiation and progression of thyroid cancer by enhancing the activation of major signaling pathways triggered by oncogenes (*NRAS* [15–17], *PTEN* [18]), forming a vicious cycle that propels its pathogenesis.

Cancers in mutation carriers usually have specific clinical characteristics, prognosis, and sensitivity to treatment. Identifying factors that increase the risk of developing a tumor is crucial. Identifying these factors will enable specific management including prevention, early detection, and treatment.

Our study aimed to identify the possible link between familial susceptibility to cancer and the level of oxidative stress in thyroid cancer patients.

## Materials and methods

### Study design and patient selection

Patients with thyroid cancer (with and without genetic predisposition) (total *n* = 60) were investigated. The study participants were treated in a Limited Liability Company (LLC) “Oncology Scientific Research Center” (Tbilisi, Georgia). The study group was collected between 2020 and 2021.

The diagnosis of thyroid pathology was based on the physical exam, ultrasound examination, fine-needle aspiration biopsy, and cytological examination and determination of the level of thyroid hormones in the blood serum.

Inclusion criteria for the study: patients with low, undifferentiated, and highly differentiated thyroid cancer as determined by cytological examination.

Exclusion criteria from the study: patients with primary multiple tumors, autoimmune thyroiditis without nodular transformation, or other thyroid gland pathology in which the presence of a nodular inclusion in the thyroid gland.

The patient’s anamnesis data were collected, including information on gender, age, smoking, alcohol consumption, and family history of cancer.

The patients included in the study are divided into three groups: Group 1 (control) - healthy people from families without cancers among I and II-degree relatives (*n* = 30), Group 2 - thyroid cancer (*n* = 30: papillary cancer − 26 patients, follicular cancer − 2 patients, medullary cancer – 1 patient, anaplastic cancer – 1 patient) patients from families with cancer aggregation, Group 3 - thyroid cancer (*n* = 30: papillary cancer − 24 patients, follicular cancer − 3 patients, medullary cancer – 2 patients, anaplastic cancer – 1 patient) patients from families without cancers among I and II-degree relatives.

The Ethics Committee of Tbilisi State Medical University approved the research design.

In patients’ blood, the thyroid hormones amount (free Triiodothyronine (fFT3), free Thyroxine (fFT4), bound Triiodothyronine (FT3), bound Thyroxine (FT4), Thyroid-stimulating hormone (TSH)), and oxidative stress intensity were investigated.

### Oxidative stress intensity

The intensity of oxidative stress in blood samples was assessed according to the total activity of the non-enzymatic antioxidant system (TAA) and the lipid peroxidation product, malondialdehyde (MDA) amount in the blood serum.

### TAA of blood serum

TAA was determined in deproteinized blood serum using the 2.2-diphenyl-1-picryl-hydrazine (DPPH)-scavenging assay, adapted by Chrzczanowicz et al. [19]. Blood serum samples (1 ml) were deproteinized by adding 3 ml of acetonitrile and centrifuging them for 10 min (4 °C, 9500 g). A supernatant was immediately collected and transferred (1 ml) into a tube, subsequently, 3 ml of DPPH was added, and the resultant solution’s absorbance was determined at wavelength 515 nm. A calibration curve was built using Gallic acid, wherein the absorbance values were interpolated and the results were expressed as Gallic acid equivalents (%).

### MDA content of blood serum

The concentration of MDA in the blood serum was measured using a Thiobarbituric acid assay [20].

### Statistical analysis

The Shapiro–Wilk test tested the null hypothesis that for each group, each study variable comes from a normally distributed population. Mean, Standard Error (Std error), and 95% Confidence Interval (CI) values ​​were calculated for each group and research variable.

One-way ANOVA was used to compare the statistical significance of differences between mean values ​​in two or more independent groups for each studied variable. Levene’s test for homogeneity of variances was used for each test variable in the compared groups.

Statistical software SPSS-10 was used for data analysis and visualization of results.

## Results

### Determination of the hormonal status in the patients’ blood

Table 1 shows the levels of thyroid hormones of study participants. The levels of free and bound forms of T4 and T3 in oncological patients’ groups (Group 2, Group 3) were not significantly different from their values ​​in the control (Group 1). there was no detected statistically significant difference between studied parameters in patients with thyroid cancer from families with cancer aggregation (Group 2) and without cancers among I and II-degree relatives (Group 3) (T4 (mean ± Std error): Group 1 (control) − 72.0 ± 2.10, Group 2–72.26 ± 1.8 (*F*_*1,2*_=0.5, *p*_*1,2*_=0.8),), Group 3–69.43 ± 1.3 (*F*_*1,3*_=2.31, *p*_*1,3*_=0.16), (*F*_*2,3*_=1.83, *p*_*2,3*_=0.63); FT4 (mean ± Std error): Group 1 (control) − 15.56 ± 0.66, Group 2–15.25 ± 0.40 (*F*_*1,2*_*=*0.8, *p*_*1,2*_*=*0.18), Group 3–15.99 ± 0.40 (*F*_*1,3*_=0.31, *p*_*1,3*_=0.88), (*F*_*2,3*_=0.24, *p*_*2,3*_=0.63); T3 (mean ± Std error): Group 1 (control) – 1.83 ± 0.05, Group 2–1.86 ± 0.06 (*F*_*1,2*_=0.76, *p*_*1,2*_=0.68), Group 3–1.89 ± 0.05 (*F*_*1,3*_=1.19, *p*_*1,3*_=0.27) (*F*_*2,3*_=0.34, *p*_*2,3*_=0.55); FT3 (mean ± Std error): Group 1 (control) – 3.66 ± 0.1, Group 2–3.70 ± 0.11 (*F*_*1,2*_=0.08, *p*_*1,2*_=0.18), Group 3–3.74 ± 0.09 (*F*_*1,3*_=0.47, *p*_*1,3*_=0.49), (*F*_*2,3*_=0.35, *p*_*2,3*_=0.55)).

The level of TSH in oncological patients’ groups (Group 2, Group 3) significantly increased compared to the control group (Group 1) (TSH (mean ± Std error): Group 1 (control) – 1.21 ± 0.12, Group 2–2.45 ± 0.11 (*F*_*1,2*_=107, *p*_*1,2*_<0.001), Group 3–2.47 ± 0.17 (*F*_*1,3*_=150, *p*_*1,3*_<0.001)), however, the difference between Group 2 and Group 3 was not recorded (*F*_*2,3*_=0.15, *p*_*2,3*_=0.80).

It is worth noting that in all studied patients, the level of thyroid hormones in the blood did not exceed the limits of clinically established norms.

**Table 1:** Levels of hormones (FT4, T4, TSH, FT3, T3) in the blood of healthy donors and patients with thyroid cancer (Mean ± Std error). Shapiro-Wilk W and p. Fisher’s F end p, and Levene’s test’s results (F, p)

Parameters	Group 1			Group 2			Group 3			ANOVA and Levene’s Test’s	Clinical norm
FT4 (mmol/l)	15.56 ± 0.66			15.25 ± 0.40			15.99 ± 0.40			F = 0.50 p = 0.61	10–22
	W = 0.97		p = 0.79	W = 0.94		p = 0.12	W = 0/96		p = 0.45	F = 13.82 p < 0.001	
T4 (nmol/l)	72.0 ± 2.10			72.26 ± 1.8			69.43 ± 1.3			F = 1.49 p = 0.32	59–160
	W = 0.95	p = 0.28		W = 0.97	p = 0.66		W = 0.97	p = 0.68		F = 16.65 p < 0.001	
TSH (mU/l)	1.21 ± 0.12			2.45 ± 0.11			2.47 ± 0.17			F = 8075 p < 0.001	0.4–4
	W = 0.94	p = 0.14		W = 0.97	p = 0.62		W = 0.96	p = 0.37		F = 5.12 p = 0.008	
FT3 (pmol/l)	3.66 ± 0.1			3.70 ± 0.11			3.74 ± 0.09			F = 0.24 p = 0.70	2.6–5.6
	W = 0.95	p = 0.30		W = 0.95	p = 0.30		W = 0.95	p = 0.32		F = 6.59 p = 0.001	
T3 (nmol/l)	1.83 ± 0.05			1.86 ± 0.06			1.89 ± 0.05			F = 0.53 p = 0.58	1.3–2.7
	W = 0.93	p = 0.051		W = 0.92	p = 0.05		W = 0.92	p = 0.05		F = 2.12 p = 0.04	

### Oxidative stress intensity in blood

Table 2 shows the content of lipid peroxidation product, MDA, and TAA in the blood serum of the enrolled patients.

**Table 2:** The MDA content and TAA in the blood serum of the studied patients. (Mean ± Std error). Shapiro-Wilk W and p. Fisher’s F end p, and Levene’s test’s results (F, p)

Parameters	Group 1			Group 2		Group 3			ANOVA and Levene’s Test’s
MDA (micromol/l)	1.43 ± 0.11			5.20 ± 0.30		4.55 ± 0.25			F = 93.28; p < 0.001
	W = 0.92	p = 0.05		W = 0.96	p = 0.36	W = 0.94		p = 0.17	F = 7.05 p = 0.001
TAA (%)	25.32 ± 0.36			16.52 ± 0.46		26.73 ± 0.92			F = 86.49 p < 0.001
	W = 0.92		p = 0.051	W = 0.92	p = 0.51	W = 0.96	p = 0.33		F = 3.82 p = 0.02

The data presented in Table 2 shows that the MDA (malondialdehyde) levels in the blood serum of patients with thyroid tumor pathology (Group 2, and Group 3) have increased by 4–5 times. However, there was no statistically significant difference between the MDA levels found in patients with thyroid cancer from families with a history of cancer (Group 2) and those without any cancers among their first and second-degree relatives (Group 3) (*F*_*2-3*_*=* 3.35; *p*_*2-3*_ *=* 0.07).

In patients with thyroid cancer from families with cancer aggregation (Group 2), the level of TAA statistically significantly decreased (*F*_*1-2*_*=*200; *p*_*1-2*_ *<* 0.001). In contrast, in patients with thyroid cancer without cancers among I and II-degree relatives (Group 3), the level of TAA did not change compared to the control *(F*_*1-3*_*=* 2.13; *p*_*1-3*_ *=* 0.15*).*

## Discussion

The molecular mechanisms underlying the etiology of cancer are not fully understood. It is thought that the initiation of cancer occurs after an accumulation of genetic alterations that results in either activation of oncogenes or inactivation of tumor suppressor genes, which lead to either cellular proliferation or abnormal programmed cell death. Reactive oxygen species (ROS) (superoxide anion (O_2_^−^), hydrogen peroxide (H_2_O_2_), and the hydroxyl radical (*OH)), generated in the cell as a result of aerobic metabolism and also as a result of inflammation, cellular stress, metabolism of exogenous compounds, at normal concentrations are intracellular signaling molecules [21, 22], serve important cellular functions, however, at high levels (oxidative stress), can participate in the initiation of several diseases, including cancer [22]. They can cause DNA damage, including mutations, deletions, gene amplification, and rearrangement. These can trigger programmed cell death, activate proto-oncogenes, and deactivate tumor suppressor genes [23].

Redox balance is regulated by the defense antioxidant system, composed of both nonenzymatic (flavonoids, glutathione, and antioxidant vitamins such as vitamins A, C, and E) and enzymatic compounds (superoxide dismutases (SOD), catalase (CAT) and glutathione peroxidase (GPX)) [21, 24], that either limits the formation of ROS or detoxify the reactive metabolites. The cellular biochemical and genetic mechanisms maintaining a balance between the relative abundance of ROS and antioxidants are complex.

ROSs have a well-established role in cell signaling: in the short term, cells adapt to oxidative stress by metabolic reprogramming (activating the antioxidant systems, maintaining ROS to homeostatic levels), and in the long term (chronic oxidative stress) - by genetic reprogramming (upregulating antioxidant gene expression and increasing cell survival) [22, 24–28]. These phenomena can have profound pathophysiological consequences [25, 29–31] - an increase in ROS can induce DNA damage, also, through the regulation of different cellular signaling pathways and nuclear factors, support the enhanced proliferation of transformed cells increase cellular growth, cell survival, contribute to genomic instability, initiate tumorigenesis and the development of cancer [26, 28, 29, 31, 32]. The antioxidant defense system can inhibit the initiation of carcinogenesis, and also affect tumor progression. The differential expression levels of each gene observed in different settings revealed a precise spatial context where redox alterations may promote genome instability or redox adaptation [33]. The production of antioxidants is diminished in various types of cancer cells. [25].

Tumor cells exhibit enhanced redox homeostasis; the level of ROS in cancer cells is phenotypically high. Sources of increased ROS production in tumor cells are associated with the production of O_2_^–^ by oncogene- or damage-stimulated mitochondria (alteration of the electron transport chain, hypoxia, or other factors) [32, 34]. Increased ROS levels in tumor cells may also result from tumor-mediated suppression of antioxidant enzymes’ genes expression (superoxide dismutase (SOD), glutathione peroxidase (GP), etc.) [29, 35], or post-translational modifications of the enzymes, exemplified by the acetylation of SOD [27, 36, 37], which confers antioxidant enzymes prooxidant properties. In addition, cancer cells may produce ROS due to the stimulation by immune cells and also, are exposed to ROS, generated by surrounding immune cells [38].

Although ROSs are considered pro-tumorigenic, high ROS levels may be cytotoxic [34, 37], and trigger aging or apoptosis of neoplastic cells [26, 34]. Cancer cells restructure their redox potential from reduced to oxidized to enhance ROS-driven proliferation. They are adapted to survive under moderate oxidative stress but avoid ROS thresholds and suppress excessive high oxidative stress that could otherwise trigger cell aging and apoptosis [34, 36] by upregulation antioxidant transcription factors or reprogram metabolism to increase the de novo synthesis of antioxidants; they also may stimulate anti-apoptotic and pro-survival pathways. Increasing antioxidant status in tumor tissue, presumably to combat high ROS burden, is generally associated with poor prognosis [39].

According to the results of our investigation, in patients with thyroid cancer, levels of T4 and T3 did not differ considerably from control levels, while TSH content increased by 90% in both groups (Group 2 and Group 3). Earlier studies did not find a significant increase in the risk of thyroid cancer related to high TSH levels or age [40]. However, recent studies have shown a strong association between high TSH levels and an increased risk of thyroid nodule malignancy [15]. Differentiated thyroid cancer cells usually express functional TSH receptors (TSHR). Stimulation of TSHR by TSH secreted in the pituitary gland increases the synthesis of H2O2, which is the substrate of thyroperoxidase in thyroglobulin iodination and thyroid hormone synthesis, contributing to the high level of oxidative stress in thyroid cancer patients. Therefore, ROS is actively generated in the malignant thyroid gland [14].

Our research indicates that the amount of MDA in the blood serum of patients diagnosed with thyroid cancer (Group 2 and Group 3) is 4–5 times higher than that of healthy individuals who participated in the study. These data are in agreement with literature data demonstrating altered lipid peroxides and antioxidants in tumor tissues of thyroid cancer patients, these alterations are increased with the progression of cancer cells toward a more differentiated phenotype [41–44]. We did not find any statistically significant difference in the levels of MDA between patients with thyroid cancer from families with a history of cancer (Group 2) and those without a history of cancer in first and second-degree relatives (Group 3). However, in patients with thyroid cancer from families with cancer aggregation (Group 2), the level of TAA decreased by 40%. In contrast, in patients with thyroid cancer without cancers among I and II-degree relatives (Group 3), the level of TAA did not change compared to control. The fact of depletion of the antioxidant system in patients with thyroid cancer from families with cancer aggregation indicates its potential role in oxidative mechanisms in malignant transformation. In vivo studies have shown that thyroid cells are more susceptible to damage resulting from oxidative stress than other organs [4].

Although the biological role of oxidative stress pathways has been extensively demonstrated, it is still unclear which and how oxidative stress genes predict bad prognosis and if their modulation is cancer-type specific. Among the genes associated with thyroid cancer BRAF, RAS, KRAS, NRAS, PTEN, and PRKAR1A genes participate in regulating oxidative metabolism. The tumor suppressor genes (PTEN, and PRKAR1A) are involved in important cellular functions, including DNA repair, regulation of transcription, ubiquitination, and cell cycle regulation [11, 13, 45]; they participate in regulating oxidative stress [46] via increasing the expression of several genes, activation antioxidant response and decrease the levels of reactive oxygen species (ROS) [47, 48], their mutation contributes to the intensification of oxidative stress. According to The Cancer Genome Atlas (TCGA) database, key genomic changes (mRNA levels) in peroxidases represented by glutathione peroxidases (GPx) and peroxiredoxins (TPx), and genes involved in the metabolism of superoxide, such as superoxide dismutase (SOD), were identified in several types of cancer. Correlative evidence suggests the involvement of principally and commonly modulated pathways of thioredoxin, superoxide dismutase, and glutathione in breast, lung, pancreatic, prostate, and colon cancers [33]. Common oncogenes mutations could increase the production of ROS through pro-inflammatory or other mechanisms to elicit strong oxidative stress and enhance the activation of major signaling pathways to promote the initiation and progression of thyroid cancer [14].

## Conclusions

Oxidative stress plays a critical role in tumorigenesis, and antioxidant/oxidant imbalance may contribute to the malignant transformation of normal tissue. In patients with familial susceptibility to cancer mutations of several genes, which are involved in the regulation of oxidative metabolism, may contribute to the disruption of the redox balance, increase the level of oxidative stress, and contribute to the development of thyroid cancer.

## Data Availability

No datasets were generated or analysed during the current study.
